# Ultrasound-Optimized Extraction and Multi-Target Mechanistic Analysis of Antioxidant and Hypoglycemic Effects of *Amomum villosum* Essential Oil

**DOI:** 10.3390/foods14162772

**Published:** 2025-08-09

**Authors:** Wenxiang Wu, Yining Liao, Lixia Wei, Xuezhen Feng, Yan Dai, Qingrong Liu, Shuzhen Feng

**Affiliations:** 1 Guangxi Key Laboratory of Research on Medical Engineering Integration and Innovation, Medical College, Guangxi University of Science and Technology, Liuzhou 545006, China; wenxiangwu@gxust.edu.cn (W.W.); liao_yining@126.com (Y.L.); w18078128606@163.com (L.W.); fengxuezhenbest@163.com (X.F.); daiyan999@foxmail.com (Y.D.); liuqingrong7173@foxmail.com (Q.L.); 2 Guangxi Industrial Technology Research Institute on Karst Rocky Desertification Control Co., Ltd., Nanning 530012, China

**Keywords:** *Amomum villosum* essential oil, ultrasound-assisted extraction, dual bioactivity, network pharmacology, molecular docking, functional foods

## Abstract

*Amomum villosum*, a medicinal and edible plant, has shown promise in improving digestive health; however, the mechanisms underlying its antioxidant and hypoglycemic effects remain unclear. This study aimed to optimize the extraction of *A. villosum* essential oil (AVEO) and elucidate its bioactive potential. Ultrasound-assisted extraction yielded 3.84% AVEO under optimal conditions. Gas chromatography–mass spectrometry combined with SwissADME analysis identified nine active components, including bornyl acetate, (−)-Spathulenol, and (−)-Pogostol. In vitro assays demonstrated potent α-glucosidase inhibition (IC_50_: 0.99 mg/mL) and strong free radical scavenging activities against 1,1-diphenyl-2-picrylhydrazyl (IC_50_: 0.87 mg/mL), hydroxyl (IC_50_: 0.18 mg/mL), and superoxide anion radicals (IC_50_: 0.01 mg/mL). A significant positive correlation was observed between its antioxidant and hypoglycemic activities. Network pharmacology identified 11 core targets involved in oxidative stress and glucose metabolism, with functional enrichment pointing to the PPAR and steroid hormone signaling pathways. Molecular docking confirmed stable binding affinities of bornyl acetate, (−)-spathulenol, and (−)-pogostol to JAK2, NCOA2, and PPARA via hydrogen bonding and hydrophobic interactions. These findings provide a mechanistic basis for the dual antioxidant–hypoglycemic effects of AVEO and support its potential application in the development of functional foods and natural therapeutics targeting metabolic disorders.

## 1. Introduction

*Amomum villosum* Lour., a medicinal food homologous plant native to tropical Asia, has traditionally been used in folk medicine across southern China and Southeast Asia to improve digestion, alleviate vomiting, and reduce abdominal distension [1,2,3]. Currently, it is also used as a natural ingredient and flavoring agent in wines, teas, candies, and cosmetics [4]. The primary constituents of *A. villosum* Lour include monoterpenes, sesquiterpenes, diterpenes, flavonoids, phenolic compounds, and polysaccharides [1]. The essential oil of *A. villosum* (AVEO), with bornyl acetate, camphor, and other terpenoids as its core components, is regarded as the primary material basis for its pharmacological activities [5]. This essential oil is listed as a quality control marker in the Chinese Pharmacopoeia, and its high content is generally regarded as an indicator of good quality [5,6].

Given its therapeutic value and significance in functional food development, the enhancement of AVEO yield represents a critical research priority. In terms of extraction technologies, traditional steam distillation offers low yields (~3% according to the Chinese Pharmacopoeia 2020, or 1.50–2.02% *v*/*w* in some studies) [5,7], and the high temperatures involved risk degrading thermolabile compounds such as bornyl acetate [5], thereby reducing bioactivity. Among emerging extraction technologies, ultrasound-assisted extraction (UAE) has shown promise as a highly efficient and selective method [8,9,10,11]. The ultrasonic cavitation theory posits that transient high pressure and localized heat generated by ultrasound selectively disrupt plant cell walls, releasing intracellular bioactive compounds while minimizing thermal degradation [12,13]. UAE aligns well with green chemistry principles and is considered a sustainable alternative to conventional methods [8]. However, optimizing ultrasound parameters is highly dependent on the properties of the plant matrix. For example, Gavahian et al. [14] demonstrated that cellulase pretreatment enhanced extraction uniformity in citrus peels; however, its applicability to lipid-rich Zingiberaceae species like *A. villosum* remains unclear. Additionally, the composition of AVEO varies markedly across different geographical origins [15], largely due to differences in terpene synthase gene expression [4]. These variations demand the development of more universal and adaptable UAE protocols. Therefore, tailored UAE optimization accounting for species-specific factors represents a critical unmet need in AVEO extraction.

Regarding the bioactivities of AVEO, partial experimental evidence has been accumulated by existing studies. Antimicrobial [5,16], antioxidant [17,18], anti-obesity [19], anti-hyperuricemic, and anti-gout arthritic activities [20] have been demonstrated for *A. villosum* extracts (not limited to essential oils). Recent in vitro and murine model studies have highlighted AVEO’s antioxidant, hypoglycemic, and anti-inflammatory characteristics [21,22], with demonstrated effects against gastric cancer [23], non-alcoholic fatty liver disease [22], alcoholic liver disease [24], and allergic rhinitis [25]. These findings underscore AVEO’s potential as a functional food additive and pharmaceutical ingredient [26]. However, the regulatory pathways through which AVEO exerts these activities remain insufficiently understood.

The multicomponent synergistic effects of plant volatile oils and their cross-pathway regulatory networks require deeper mechanistic investigation. Network pharmacology and molecular docking have recently emerged as powerful tools for analyzing the multi-target effects of plant-derived compounds [26,27]. Emerging evidence indicates that antioxidant effects in A. villosum stem–leaf extracts may involve novel bioactive compounds that stabilize PGAM5 binding through residue-specific hydrogen bonds (Lys93/Arg214) [26]. Reference pathways regulating oxidative stress in other essential oils include γ-aminobutyric acidergic synapses [28], AGE-RAGE [29], calcium signaling [30], and PI3K/AKT/mTOR cascades [31], with the latter recognized as a conserved hypoglycemic pathway [31]. While multicomponent bioactivities attract growing interest, synergistic mechanisms remain less explored than single-compound effects. It has been reported that activation of the Nrf2-Keap1 pathway constitutes an established antioxidant mechanism with a demonstrated capacity to ameliorate diabetes and its complications [32]. Nevertheless, it remains unclear whether AVEO exerts its effects via this pathway. Furthermore, the precise mechanisms underlying the synergistic actions of AVEO components in oxidative stress and glucose metabolism are still poorly understood.

To address these knowledge limitations, this study proposes an integrated “process optimization-functional validation-mechanistic elucidation” framework. First, a Box–Behnken response surface methodology was used to optimize ultrasound parameters (extraction time, ultrasonic power, and solvent ratio) for maximum yield and component retention, with validation across samples of different origins and harvest years. Second, α-glucosidase inhibition and radical scavenging assays were conducted to quantify the hypoglycemic and antioxidant capacities of AVEO. Finally, gas chromatography–mass spectrometry (GC-MS) analysis, network pharmacology, and molecular docking were used to construct a “component–target–pathway” network to identify key bioactive compounds and their molecular targets, elucidating AVEO’s metabolic regulatory mechanisms. We hypothesized that (1) UAE enhances AVEO extraction efficiency; (2) AVEO exhibits potent hypoglycemic and antioxidant activities; and (3) the hypoglycemic–antioxidant synergy may be mediated by metabolic or oxidative stress-related pathways such as PI3K/AKT and Nrf2-Keap1. The novel integration of process optimization, bioactive profiling, and network pharmacology-based mechanistic investigation represents the core innovation of this study, addressing both extraction applicability and underlying mechanistic insights. The findings of this study provide a scalable technical solution for AVEO extraction and a mechanistic basis for the functional development of *A. villosum* as a natural food additive.

## 2. Materials and Methods

### 2.1. Plant Material

Fruits of *Amomum villosum* Lour. were collected in 2023 and 2022 from cultivation sites in Baise (23.90° N, 106.62° E) and Long’an (23.18° N, 107.45° E), Guangxi, China, respectively. The samples were provided by Guangxi Botanical Garden of Medicinal Plants. Specimens were authenticated by Dr. Hu Renchuan (Guangxi Institute of Chinese Medicine) and Dr. Huang Yuan (Guangxi Botanical Garden of Medicinal Plants). Each sample consisted of mixed fruits collected from three randomly 1 × 1 m quadrats at the cultivation base. The samples were freeze-dried for 72 h until constant weight, ground, sieved through a 40-mesh screen, vacuum-sealed, and stored at –20 °C until analysis. They were labeled as Amomum1, Amomum2, Amomum3, and Amomum4 (Table 1).

### 2.2. Chemicals and Reagents

α-Glucosidase (EC 3.2.1.20, activity ≥ 10 U/mg), 1,1-diphenyl-2-picrylhydrazyl (DPPH), and salicylic acid were purchased from Sigma-Aldrich, St. Louis, MO, USA. Acarbose (purity ≥ 98%) was obtained from Shanghai Yuanye Bio-Technology Co., Ltd., Shanghai, China.

### 2.3. Experimental Methods

#### 2.3.1. Optimization of Essential Oil Extraction

- Extraction procedure: Essential oil was extracted using a customized ultrasound-essential oil co-extraction system (Model KQ5200DV, Shanghai Precision Instrument Co., Shanghai, China) following Method A of the Chinese Pharmacopoeia (2020 Edition, Part IV). Briefly, 15.0 g of *A. villosum* powder was mixed with ultrapure water at a preset solvent-to-material ratio and subjected to UAE at 25 ± 1 °C. The extraction yield (Y, %) was calculated using the formula:
  (1) $$Y(\%)=\frac{V}{m}\times 100$$
  where V is the volume of extracted essential oil (mL), and m is the mass of the powder sample (g).
- Single-factor experiments: To identify optimal conditions, the following parameters were evaluated individually:
  1. Ultrasound time: 5, 10, 15, 20, and 25 min under fixed conditions (200 W, 1:20 g/mL).
  2. Solvent-to-material ratio: 1:15, 1:20, 1:25, 1:30, and 1:35 g/mL after, optimizing the ultrasound time (15 min).
  3. Ultrasound power: 120, 160, 200, 240, and 280 W (using the optimal time and ratio).
  4. Each experiment was performed in triplicate.
- Box–Behnken response surface design: Three independent variables—ultrasound time (A: 10, 15, 20 min), solvent-to-material ratio (B: 1:20, 1:25, 1:30 g/mL), and ultrasound power (C: 160, 200, 240 W)—were optimized using Design-Expert 13.0 software (Stat-Ease, Inc., Minneapolis, MN, USA). The design included 17 runs with 5 center points. A quadratic polynomial model was used for data fitting.

#### 2.3.2. Hypoglycemic and Antioxidant Activity Assays

The residual moisture in the extracted AVEO was initially dehydrated using anhydrous sodium sulfate, followed by filtration through a 0.22 μm membrane for subsequent use.

- α-Glucosidase Inhibition:

Adapted from Yang et al. [33], 25 μL of essential oil ethanol solution (0.5, 1.0, 1.5, 2.0, 2.5 mg/mL) and 25 μL of α-glucosidase (1 U/mL in phosphate-buffered saline, pH 6.8) were preincubated in a 96-well plate at 37 °C for 10 min. Then, 50 μL of 2.5 mM p-nitrophenyl-α-D-glucopyranoside was added, and the mixture was incubated for 30 min. The reaction was terminated by adding 100 μL of 0.2 M Na_2_CO_3_. Absorbance was measured at 405 nm (Multiskan GO, Thermo Fisher Scientific Co., Ltd., Waltham, MA, USA). The inhibition rate was calculated as follows:

(2) $$Inhibition(\%)=(1-\frac{A_{1}-A_{3}}{A_{2}-A_{4}})\times 100$$

where A_1_ is the absorbance of the sample (AVEO) with enzyme; A_2_ is the control (buffer + enzyme); A_3_ is the background (AVEO + buffer); and A_4_ is the blank (buffer only). Acarbose was used as the positive control to validate the assay system and provide a reference for α-glucosidase inhibitory activity.

- Free Radical Scavenging Assays:
  1. DPPH Assay: 1 mL of AVEO solution (0.2–1.0 mg/mL) was mixed with 1 mL of 0.1 mM DPPH ethanol solution and incubated in the dark for 30 min. Absorbance was measured at 517 nm [34].
  2. Hydroxyl Radical (·OH) Assay: 1 mL of AVEO solution was mixed with 0.5 mL of 6 mM FeSO_4_ and 0.5 mL of 8.8 mM H_2_O_2_. After 10 min, 0.5 mL of 6 mM salicylic acid was added, and absorbance was measured at 510 nm after 30 min at 37 °C [35].
  3. Superoxide Anion (O_2_^−^·) Assay: 0.5 mL of AVEO solution was added to 1.5 mL of 50 mM Tris-HCl (pH 8.2) and incubated at 25 °C for 20 min. Then, 0.5 mL of 3 mM pyrogallol (in 10 mM HCl) was added. After 5 min, the reaction was terminated with HCl, and absorbance was measured at 320 nm [36].

Scavenging activity (%) was calculated as follows:

(3) $$Scavenging(\%)=(1-\frac{A_{1}-A_{2}}{A_{0}})\times 100$$

where A_1_ is the sample; A_2_ is the background (radical source replaced with buffer); and A_0_ is the blank (sample replaced with buffer). Ascorbic acid (vitamin C) was used as the positive control to validate the radical scavenging assay system and serve as a reference for antioxidant activity.

#### 2.3.3. GC-MS Analysis of AVEO Components

The samples were dried with anhydrous sodium sulfate and filtered through a 0.22 μm filter membrane. GC-MS was conducted using an Agilent 7890A–5975C system (Agilent Technologies, Inc., Santa Clara, CA, USA) equipped with an HP-1ms capillary column (60.0 m × 250 μm × 0.25 μm). The initial oven temperature was set to 60 °C (held for 1 min), ramped to 130 °C at 5 °C/min (held for 1 min), then to 160 °C at 2 °C/min (no hold, i.e., 0 min), and finally to 230 °C at 5 °C/min. Helium was used as the carrier gas at a flow rate of 1.2 mL/min. Mass spectrometry conditions: EI source (70 eV), ion source temperature 230 °C, scan range 35–500 *m*/*z*. Compounds were identified by matching mass spectra against the NIST 20 library (match factor > 85). Relative content (C_i_) was determined by peak area normalization and was retrieved from the system-generated report.

(4) $$C_{i}=\frac{A_{i}}{A_{1}+A_{2}\cdots +A_{i}+\cdots +A_{n}}\times 100\%$$

where the following definitions apply: C_i_—percentage content of the *i*-th identified compound (%); A_i_—chromatographic peak area of the *i*-th compound; n—total number of identified compounds.

### 2.4. Data Analysis

#### 2.4.1. Validation of Response Surface Model

A quadratic regression model was developed using Design-Expert 13.0 (Stat-Ease, Inc., Minneapolis, MN, USA) to relate extraction yield (Y) to ultrasound time (A), solvent-to-material ratio (B), and ultrasound power (C). Model significance was assessed using analysis of variance (ANOVA), including *p*-values, lack of fit, coefficient of determination (R^2^), and adjusted R^2^. Residual normality was verified using the Shapiro–Wilk test (SPSS 25.0, IBM Corp., Armonk, NY, USA). All experiments were conducted independently in triplicate.

#### 2.4.2. Statistical Analysis of Bioactivity Data

Data from α-glucosidase inhibition and radical scavenging assays were analyzed using one-way ANOVA, followed by Tukey’s post hoc test (*p* < 0.05). Dose–response curves and IC_50_ values (95% confidence interval [CI]) were generated using a four-parameter logistic regression model (GraphPad Prism 10.0, GraphPad Software, LLC, San Diego, CA, USA). Pearson correlation and heat maps were used to visualize associations between antioxidant and hypoglycemic activities.

#### 2.4.3. Network Pharmacology Analysis

Candidate compounds identified via GC-MS were screened for high gastrointestinal absorption—a parameter indicating favorable pharmacokinetic properties, using SwissADME analysis (http://www.swissadme.ch/index.php, accessed on 30 May 2025) [36,37]. Target prediction was conducted using SwissTargetPrediction (http://www.swisstargetprediction.ch/, accessed on 30 May 2025) [38], and gene names were standardized using UniProt (https://www.uniprot.org/, accessed on 30 May 2025) [39]. Disease-related genes for oxidative stress and diabetic nephropathy were obtained from GeneCards (https://www.genecards.org/, accessed on 31 May 2025) [40] and OMIM (https://www.omim.org/, accessed on 31 May 2025) [41].

Common targets between *A. villosum* components and antioxidant–hypoglycemic genes were identified using Venny 2.1.0. A “compound–target–disease” network was constructed in Cytoscape 3.9.1 (Cytoscape Consortium, San Diego, CA, USA), with node degree (Degree) used to identify core targets. Higher degree values indicated greater functional importance within the network [42].

Pathway Enrichment and Protein–Protein Interaction Network: DAVID (https://david.ncifcrf.gov/, accessed on 2 June 2025) [43] was used to perform gene ontology (GO) functional enrichment and Kyoto Encyclopedia of Genes and Genomes (KEGG) pathway analyses on intersection targets. The top 10 pathways (*p* < 0.05) related to antioxidant and hypoglycemic activities were visualized. A protein–protein interaction (PPI) network for the compound-disease intersection was generated using STRING (https://cn.string-db.org/, accessed on 3 June 2025) [44], and core modules were identified using the MCODE plugin.

Molecular Docking Validation: Key bioactive compounds and target proteins were subjected to molecular docking. Receptor structures with a resolution ≤ 2.5 Å were retrieved from the PDB database (https://www.rcsb.org/, accessed on 5 June 2025) [45], and three-dimensional of ligands structures were obtained from PubChem (https://pubchem.ncbi.nlm.nih.gov/, accessed on 6 June 2025) [46]. Ligands and water molecules were removed using PyMOL (Schrödinger, LLC, New York, NY, USA). Docking simulations were performed using AutoDockTools-1.5.7 (The Scripps Research Institute, La Jolla, CA, USA), with docking boxes set to enclose the entire binding pocket. Lower binding energy is generally associated with strong ligand–receptor interactions [47]. A binding affinity ≤ −5.0 kcal/mol is considered indicative of strong binding potential [48,49], representing as an effective interaction between the ligand and the target compound [50]. The results were visualized using Discovery Studio 4.5 (Dassault Systèmes BIOVIA, San Diego, CA, USA).

## 3. Results

### 3.1. Optimization of AVEO

**Figure 1:**
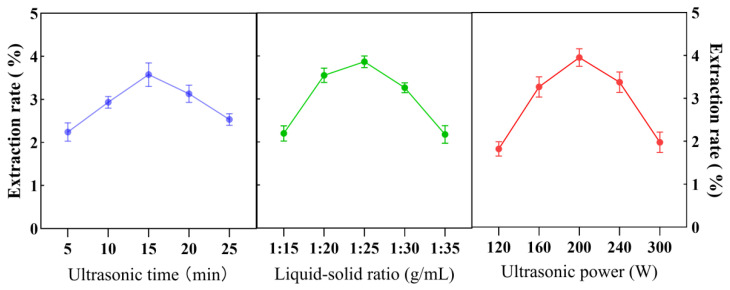
Results of single-factor experiments on AVEO (*A. villosum* essential oil). Influence of ultrasonic time, solvent-to-material ratio, and ultrasonic power on the extraction yield of AVEO.

Under single-factor experimental conditions, the optimal parameters for essential oil extraction were identified as follows: ultrasound time of 15 min, solvent-to-material ratio of 1:25 g/mL, and ultrasound power of 200 W (Figure 1).

A quadratic polynomial regression model was developed based on 17 Box–Behnken experimental runs (Table S1), describing the relationship among ultrasound time (A), solvent-to-material ratio (B), ultrasound power (C), and essential oil yield (Y), as follows:

(5) Y = 3.77 − 0.0075A + 0.1075B + 0.0325C − 0.1175AB + 0.1025AC − 0.0675BC − 0.3808A^2^ − 0.2457B^2^ − 0.4257C^2^

ANOVA (Table 2) indicated that the model was statistically significant (*p* = 0.0011), with a non-significant lack-of-fit term (*p* = 0.9379), confirming that the model errors primarily originated from random factors rather than systematic bias. The high correlation coefficient (R^2^ = 0.95) validated the reliability of the experimental design and the accuracy of the model.

**Figure 2:**
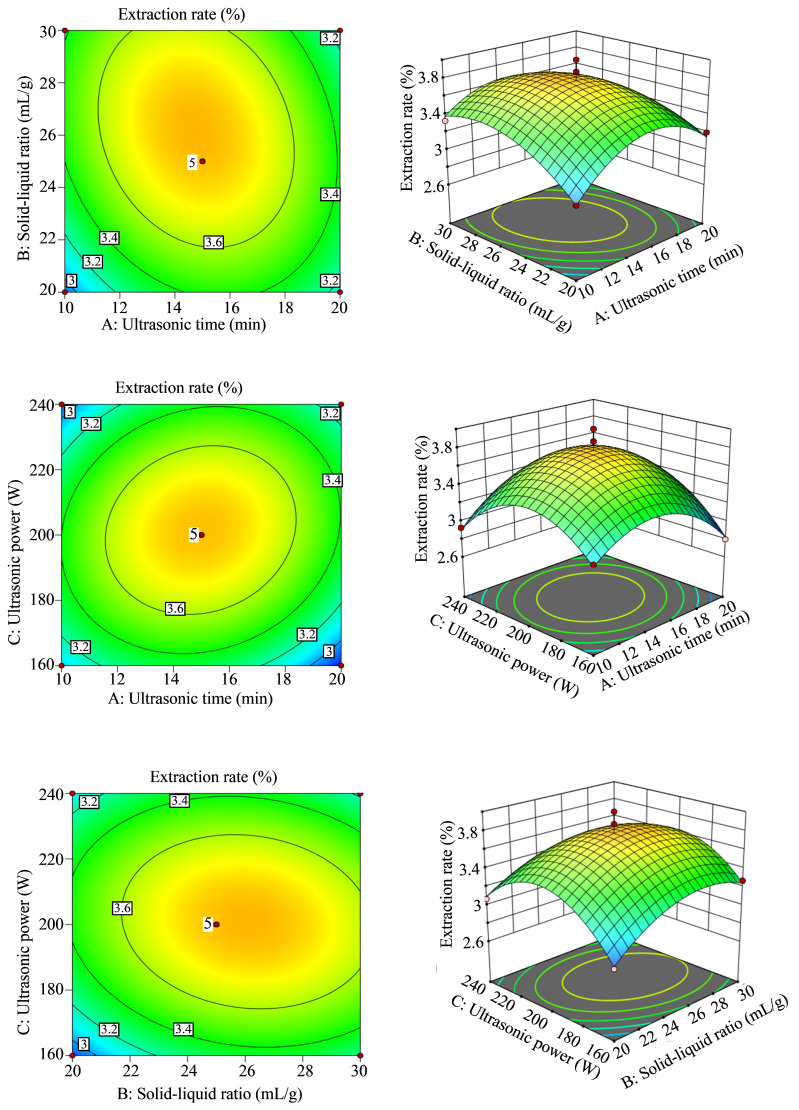
Response surface plots for extraction optimization. Three-dimensional surface plots showing interaction effects among ultrasonic time, solvent-to-material ratio, and ultrasonic power on AVEO yield, based on the Box–Behnken design.

Standardized effect analysis (Figure 2) ranked the key influencing factors in the following order: solvent-to-material ratio > ultrasound power > ultrasound time.

The predicted optimal conditions from the model were 15.00 min ultrasound time, 1:26.13 g/mL solvent-to-material ratio, and 200.59 W ultrasound power, yielding a theoretical essential oil output of 3.79%.

A validation experiment using the adjusted ultrasound power (200 W) achieved an actual oil yield of 3.84 ± 0.08% (RSD = 1.95%, *n* = 3), representing only a 1.3% deviation from the predicted value. Additional validation with Amomum 2–4 yielded consistent results, with an average essential oil yield of 3.47 ± 0.10%, confirming the model’s applicability and stability across samples.

### 3.2. Hypoglycemic and Antioxidant Properties of AVEO

#### 3.2.1. α-Glucosidase Inhibitory Activity

**Figure 3:**
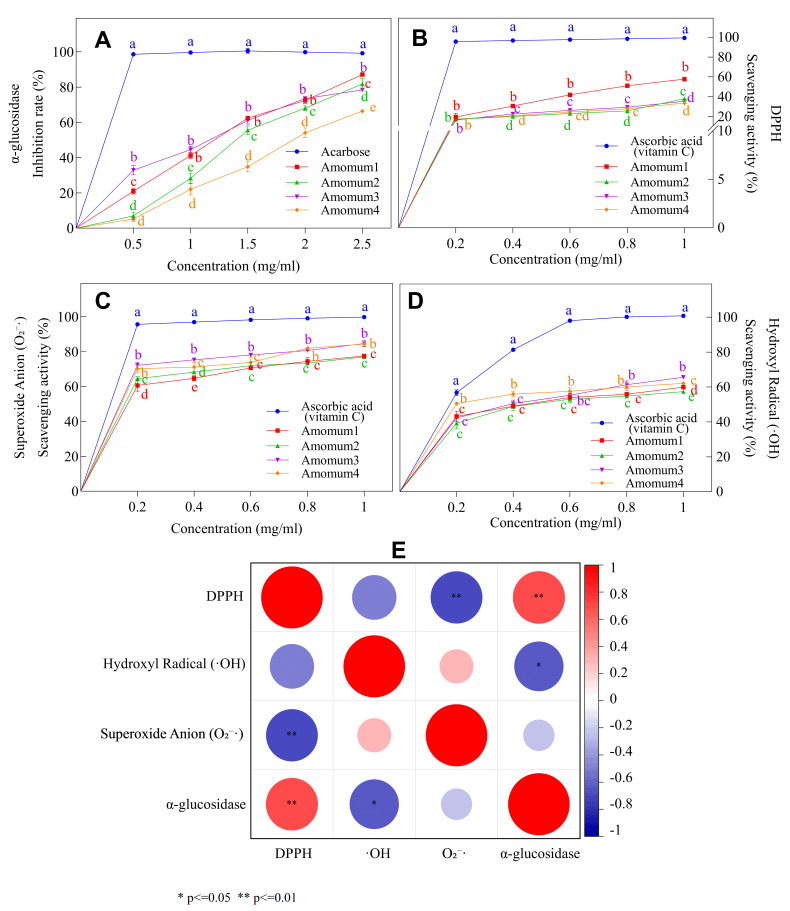
Antioxidant and hypoglycemic activities of AVEO, including: (**A**) α-glucosidase inhibition; (**B**) 1,1-diphenyl-2-picrylhydrazyl (DPPH) scavenging activity; (**C**) superoxide anion (·O_2_^−^) scavenging activity; (**D**) hydroxyl radical (·OH) scavenging activity; (**E**) heatmap showing the correlation between free radical scavenging capacity and hypoglycemic activity. Different lowercase letters indicate statistically significant differences between samples at the same concentration (LSD test, *p* < 0.05), while identical letters indicate no significant difference.

AVEO exhibited a dose-dependent inhibitory effect on α-glucosidase (Figure 3A). Among the tested samples, Amomum3 demonstrated the strongest inhibitory activity, with an IC_50_ value of 0.99 ± 0.05 mg/mL, significantly outperforming the other congeners (IC_50_ = 1.10–1.87 mg/mL). Notably, Amomum1 achieved the highest inhibition rate of 87.07 ± 1.23% at a concentration of 2.5 mg/mL, comparable to that of the clinical hypoglycemic drug acarbose. These findings suggest that AVEO, particularly Amomum1 and Amomum3, holds promise as a natural sugar-lowering ingredient for functional food applications.

#### 3.2.2. Antioxidant Capacity

AVEO exhibited strong antioxidant activity by scavenging DPPH radicals, hydroxyl radicals (·OH), and superoxide anions (·O_2_^−^) (Figure 3B–D). At a concentration of 1 mg/mL, the scavenging rates were 57.79%, 84.59%, and 65.61% for DPPH, ·OH, and ·O_2_^−^, respectively. Amomum1 exhibited the strongest DPPH radical scavenging activity (IC_50_ = 0.78 ± 0.02 mg/mL), while Amomum3 showed the highest ·O_2_^−^ scavenging activity (IC_50_ = 0.012 ± 0.01 mg/mL). Conversely, Amomum4 demonstrated superior ·OH scavenging ability (IC_50_ = 0.18 ± 0.04 mg/mL), comparable to the standard antioxidant ascorbic acid (vitamin C). Overall, Amomum3 was particularly effective in scavenging both ·OH and ·O_2_^−^.

#### 3.2.3. Functional Synergy Analysis

Spearman correlation analysis (Figure 3E) revealed a significant positive correlation between α-glucosidase inhibitory activity and DPPH scavenging capacity (r = 0.82, *p* < 0.01), as well as a significant negative correlation with ·OH scavenging capacity (r = −0.65, *p* < 0.05). These relationships suggest that the inhibitory and antioxidant effects of AVEO are mediated by overlapping bioactive compounds responsible for both α-glucosidase inhibition and DPPH radical scavenging. The negative correlation between its α-glucosidase inhibitory activity and ·OH scavenging capacity may indicate substrate competition or spatial antagonism at shared binding sites. Collectively, these findings support the potential of AVEO as a dual-functional food additive possessing both antioxidant and glucose-regulating properties.

### 3.3. Composition Analysis of AVEO

**Figure 4:**
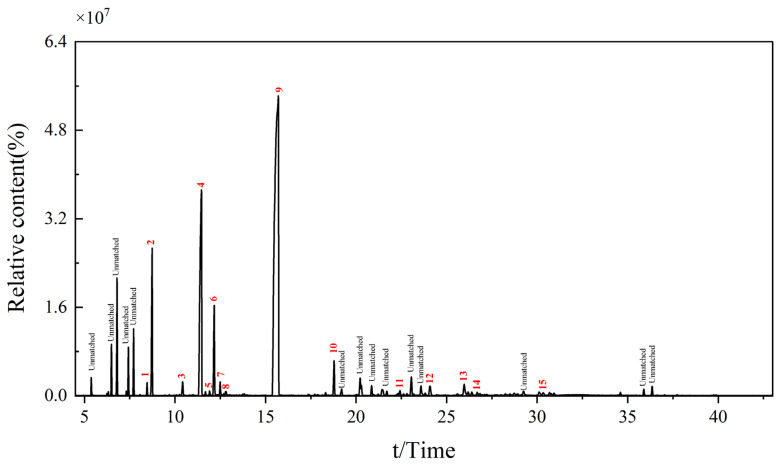
Gas chromatography–mass spectrometry (GC-MS) total ion chromatogram (TIC) of AVEO. The chromatographic profile shows the total ion counts of volatile compounds in AVEO, as identified using GC–MS. Peaks labeled with numbers correspond to the compounds listed in Table 3, which account for 81.94% of the total relative content. Peaks marked as “Unmatched” represent components with a match factor < 85 (based on the NIST 20 library), which are not commonly reported in existing literature for *Amomum* species. Unmarked peaks represent minor constituents (<0.1% relative content) that were excluded from analytical consideration.

GC-MS analysis of Amomum3 *AVEO* (Figure 4) identified 15 characteristic components (Table 3), with their relative contents quantified using chromatographic peak area normalization. The predominant compounds included bornyl acetate (52.1%), (+)-2-bornanone (15.91%), cyclohexane-1-butenylidene (5.21%), and 5,5-dimethyl-1,3-hexadiene (3.52%).

### 3.4. Network Pharmacology Analysis of the Antioxidant and Hypoglycemic Mechanisms of AVEO

#### 3.4.1. Screening of Candidate Compounds and Potential Targets

Nine candidate compounds with high gastrointestinal absorption were identified from AVEO using GC-MS and SwissADME screening (Table 3, A1–A9). These included primarily monoterpenoid—(+)-2-bornanone, bornyl acetate, linalool, isoborneol, terpinen-4-ol, and α-terpineol—and sesquiterpenoids, such as (–)-spathulenol, (–)-pogostol, and α-bisabolol.

Following UniProt standardization, 127 well-characterized target genes were mapped to these compounds. Through multi-database integration (see Methods), 5014 antioxidant-related and 4049 hypoglycemic-related genes were identified.

**Figure 5:**
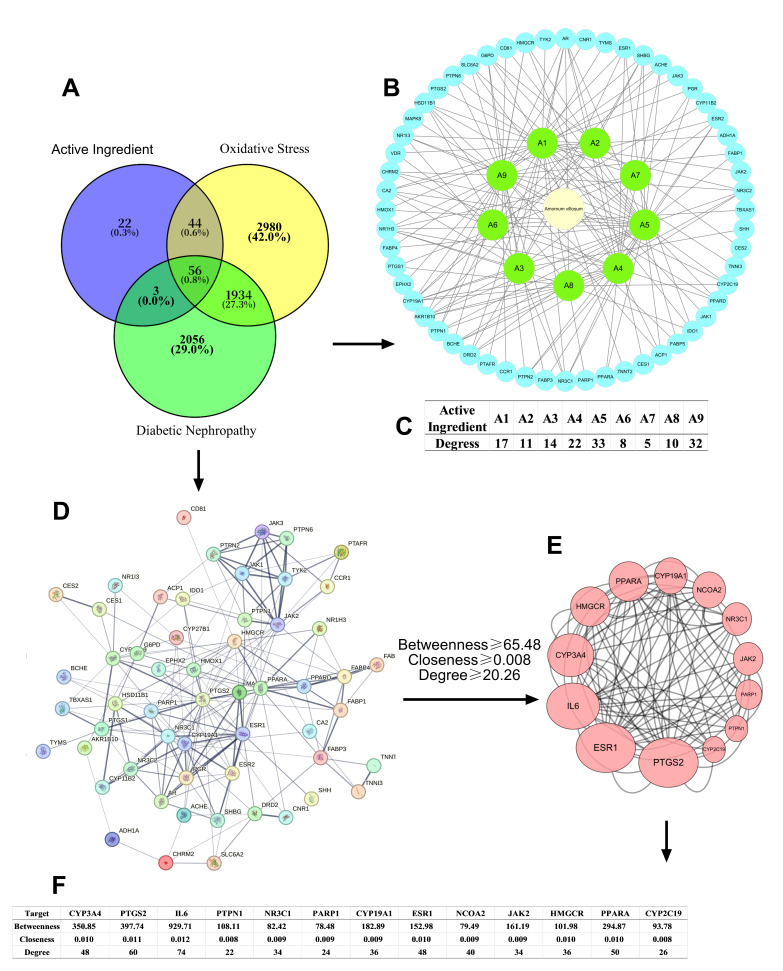
Network pharmacology of AVEO: (**A**) targets of active components in *Amomum villosum* and antioxidant–hypoglycemic-related genes; (**B**) “compound–disease–target gene” network; (**C**) degree values of each compound in the “compound–disease–target gene” network; (**D**) protein–protein interaction (PPI) network; (**E**) core targets; (**F**) data related to core targets.

A total of 56 overlapping targets were identified among the component-related, antioxidant, and hypoglycemic gene sets (Figure 5A). An “*A. villosum*-Active Component-Disease-Target” network was constructed using Cytoscape 3.9.0 (Figure 5B,C). All nine compounds exhibited a high degree of centrality, indicating their central roles as regulators of antioxidant and hypoglycemic pathways.

A PPI network of shared targets was established (Figure 5D), consisting of 56 proteins interconnected by 199 edges (average node degree: 7.11; average local clustering coefficient: 0.50). Enrichment analysis showed statistically significant functional involvement (*p* < 1.0 × 10^−16^), suggesting critical functions in antioxidant and hypoglycemic processes. Core genes identified via topological analysis (Betweenness unDir, Closeness unDir, Degree metrics exceeding mean values) included CYP3A4, PTGS2, IL6, PTPN1, NR3C1, PARP1, CYP19A1, ESR1, NCOA2, JAK2, HMGCR, PPARA, and CYP2C19 (Figure 5E), underscoring their pivotal regulatory roles in both antioxidant and hypoglycemic responses. Node size was scaled according to the degree value. Key values of the core targets are shown in Figure 5F.

#### 3.4.2. Functional Enrichment

**Figure 6:**
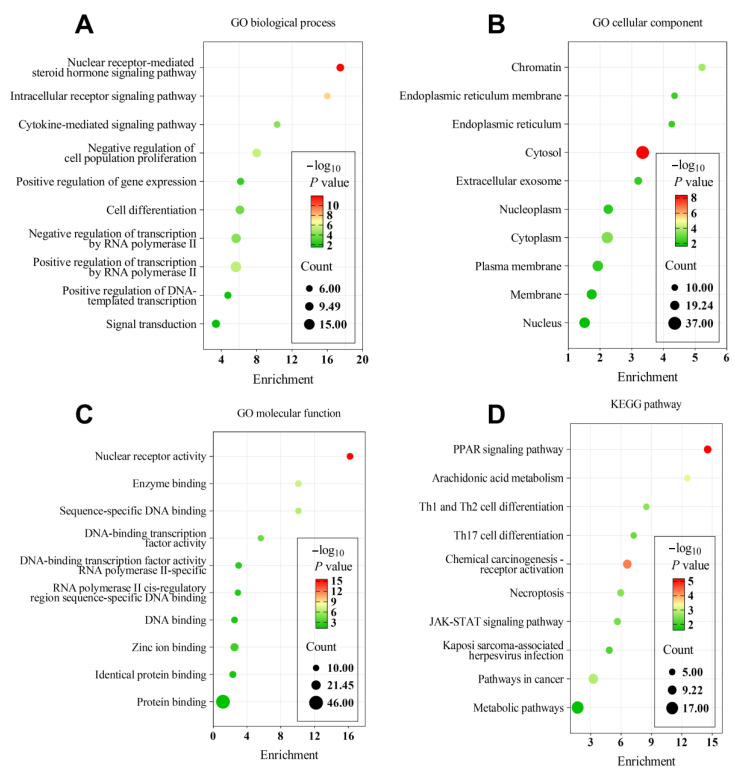
Functional enrichment analysis of AVEO target genes with (**A**) the gene ontology (GO) biological process, (**B**) the GO cellular component, (**C**) the GO molecular function, and (**D**) the KEGG pathway. Abbreviations: PPAR, peroxisome proliferator-activated receptor; JAK-STAT, Janus kinase–signal transducer and activator of transcription; GO, gene ontology; KEGG, Kyoto Encyclopedia of Genes and Genomes; RNA, ribonucleic Acid; DNA, deoxyribonucleic Acid.

Functional enrichment analyses of the 56 intersecting target genes associated with the antioxidant and hypoglycemic activities of AVEO identified 123 significantly enriched biological processes, 25 cellular components, and 53 molecular functions (*p* < 0.05). The top 10 terms most closely associated with oxidative stress and glucose regulation in each category were visualized (Figure 6A–C). GO biological process analysis (Figure 6A) revealed significant enrichment in nuclear receptor-mediated steroid hormone signaling and intracellular receptor signaling pathways. GO cellular component analysis (Figure 6B) identified the chromatin, endoplasmic reticulum membrane, and cytosol as the primary locations. GO molecular function analysis (Figure 6C) showed significant enrichment in nuclear receptor activity, enzyme binding, and protein binding.

KEGG pathway analysis (Figure 6D) identified 18 significantly enriched pathways (*p* < 0.05), with the peroxisome proliferator-activated receptor (PPAR) signaling pathway, arachidonic acid metabolism, and general metabolic pathways being the most critical. These findings provide molecular-level evidence that the essential oil exerts dual antioxidant and hypoglycemic effects through synergistic regulation of nuclear hormone signaling, lipid metabolism, and oxidative stress homeostasis.

### 3.5. Molecular Docking Validation

**Figure 7:**
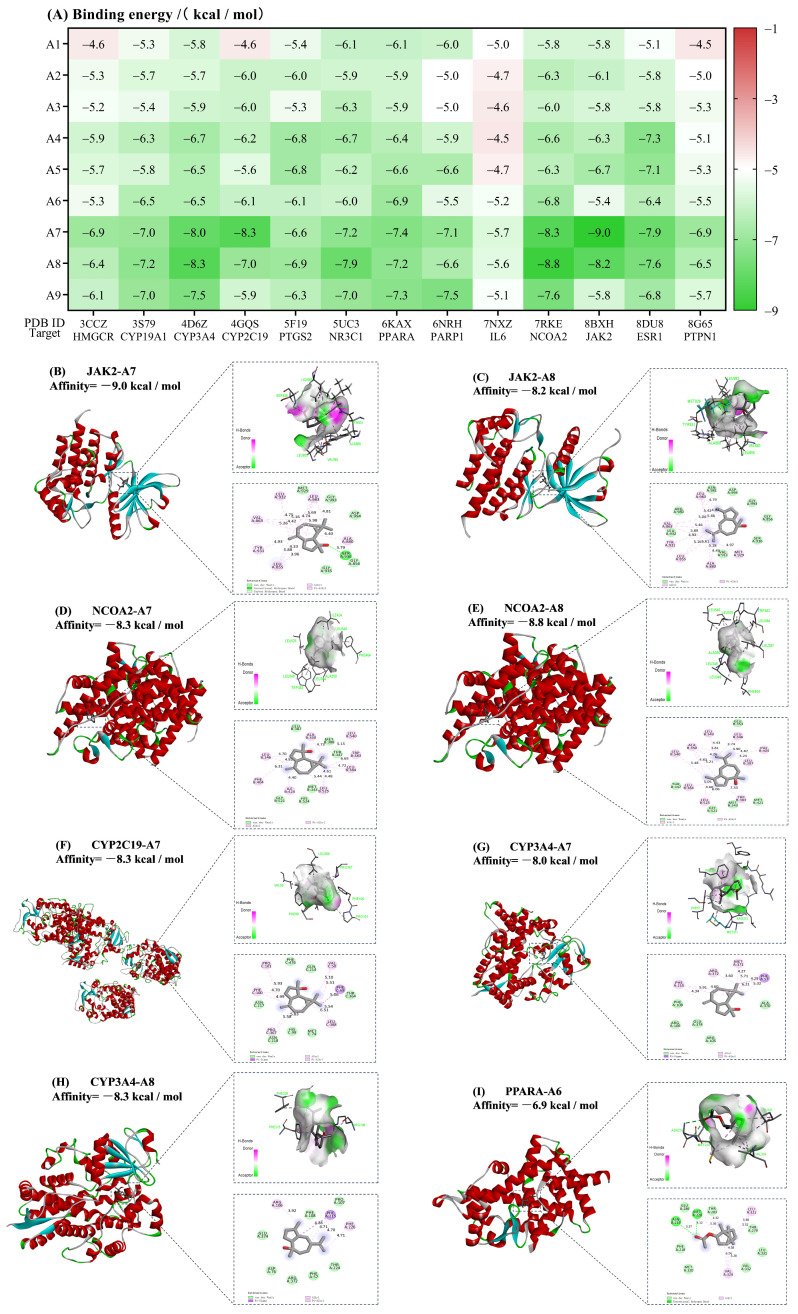
Molecular docking results of AVEO compounds with core targets: (**A**) heatmap of binding energies between AVEO active components and protein targets; (**B**–**H**) representative docking poses showing binding interactions (hydrogen bonding and hydrophobic forces) with energy values < −8 kcal/mol; (**I**) binding conformation of bornyl acetate (dominant constituent, 52.10%) with *PPARA*.

Molecular docking studies revealed that compounds A4–A9 in AVEO (e.g., terpinen-4-ol, α-terpineol, bornyl acetate, (−)-spathulenol, (−)-pogostol, α-bisabolol) exhibited significant multi-target binding activities against core targets involved in both antioxidant and hypoglycemic pathways (e.g., CYP3A4, CYP2C19, PTGS2, NR3C1, PPARA, NCOA2, JAK2, ESR1) (Figure 7A). Most compounds exhibited binding energies below −6.0 kcal/mol across all targets, indicating relatively stable binding energies. The lowest binding energy was observed for (−)-spathulenol binding to JAK2 (−9.0 kcal/mol), stabilized by hydrogen bonds (e.g., Ser936) and hydrophobic interactions (e.g., Tyr930, Leu855, Leu982, Leu983, Ala880, and Val863) (Figure 7B). Similarly, (−)-pogostol showed stable binding to NCOA2 (−8.8 kcal/mol), primarily mediated through hydrophobic interactions (e.g., Leu346, Leu349, Leu384, Leu387, Leu525, Ala350, Trp383, and Phe404) (Figure 7E). In addition to NCOA2 and JAK2, both (−)-spathulenol and (−)-pogostol also stably bound to CYP3A4 and CYP2C19 via hydrophobic interactions, with binding energies below −8.0 kcal/mol (Figure 7C,D,F–H). Notably, bornyl acetate (52.10% by GC-MS) bound stably to all core targets with binding energies below −5.2 kcal/mol. It showed the strongest interaction with PPARA (−6.9 kcal/mol) through hydrogen bonds (e.g., Asn219, Met220) and hydrophobic contacts (e.g., Leu321, Val324) (Figure 7I). Collectively, these computational findings suggest that the key bioactive constituents’ AVEO may synergistically interact with multiple targets through strong binding affinities, providing preliminary mechanistic support for its potential dual antioxidant–hypoglycemic effects.

## 4. Discussion

### 4.1. Significant Optimization of AVEO Ultrasonic Extraction Process

The UAE protocol established in this study achieved synergistic optimization of yield (3.84%) and retention of bioactive compounds (52.10%) in AVEO (Figure 1 and Figure 4). Compared with previous reports stating that *A. villosum* contains 1.7–3.0% essential oil with bornyl acetate (its major component) accounting for 5–47% [4,51], our results show superior performance in both yield and bioactive compound retention. These findings not only strongly support the first hypothesis of this study but also fully validate the significant value of UAE technology in plant essential oil extraction. Model predictions closely matched the experimental outcomes with only a 1.3% deviation, indicating strong model reliability. Regression analysis revealed nonlinear regulatory effects of ultrasonic parameters (time, power, solvent-to-material ratio) on extraction yield, with the solvent-to-material ratio (parameter B) identified as the critical factor. This aligns with previous research demonstrating that an optimized solvent-to-material ratio enhances essential oil yield by improving solvent penetration efficiency [52], while ultrasonic cavitation-induced microjets accelerate cell wall disruption to release lipophilic compounds [12,53]. Despite known variations in plant metabolite profiles due to geographical and environmental factors [54,55], the optimized UAE protocol consistently yields 3.47 ± 0.10% across multi-year and multi-region samples, indicating its robustness against raw material variability. Compared to traditional steam distillation (4–6 h) and supercritical CO_2_ extraction [51], UAE reduced extraction time to just 1 h [12] while increasing bornyl acetate retention by 5.10% (Figure 4). These advantages likely result from two key ultrasonic mechanisms: (1) enhanced mass transfer efficiency through cavitation [56,57] and (2) localized high-temperature/high-pressure microenvironments that selectively protect thermolabile terpenes [58,59]. Notably, excessive ultrasonic power triggered a “threshold ultrasound” phenomenon, where reaction efficiency decreased despite increased energy input [60]. Our study observed this effect, and optimized parameters (200 W, 15 min) were selected to maximize yield without compromising compound stability. The high predictive accuracy (R^2^ = 0.95) confirms the reliability of response surface methodology for complex process optimization [61], providing a paradigm for the green extraction of natural products.

### 4.2. AVEO Exhibited Excellent Hypoglycemic and Antioxidant Activities

In vitro assays demonstrated that AVEO (Amomum3) exhibited potent α-glucosidase inhibitory activity (IC_50_ = 0.99 ± 0.05 mg/mL), with 87.07% maximum inhibition at high concentrations (Figure 3A). Although significantly less potent than that of the clinical drug acarbose, its activity was still comparable. At 1 mg/mL, ·OH and O_2_^−^· scavenging capacities reached 84.59% and 65.61%, respectively (Figure 3C,D), with O_2_^−^· scavenging approaching the reference levels of vitamin C. These findings support the second hypothesis regarding AVEO’s dual bioactivity.

Regarding the hypoglycemic activity of AVEO, our findings are consistent with previous studies, although the inhibition rate observed in our experiments was higher than the range (31.99–62.58%) reported by Kim [62]. This enhanced activity may be attributed to the high bornyl acetate content (52.1%) in AVEO, as terpene-rich essential oils are increasingly recognized as potential antidiabetic agents targeting carbohydrate-metabolizing enzymes [63]. Consistent with our findings, in vivo studies have documented pancreatic β-cell protection and amelioration of ultrastructural changes in diabetic models [62,64,65], while reviews have acknowledged its hypoglycemic potential [1]. Meanwhile, we found that the α-glucosidase inhibitory activity of AVEO was superior to that of several reported natural hypoglycemic plants. These include cinnamon extract (IC_50_ = 1.15 mg/mL [66]), bitter melon terpenoids (IC_50_ = 1.60 mg/mL [67]), hot-pressed peanut meal protein hydrolysates (IC_50_ = 5.63 mg/mL [68]), *Dianthus basuticus* saponins (IC_50_ = 3.80 mg/mL [69]), and *Citrus paradisi* peel extract (IC_50_ = 1.23 mg/mL [70]). Here, the IC_50_ value of Amomum3 was comparable to that of acarbose (IC_50_ = 0.75 mg/mL), a commonly used clinical α-glucosidase inhibitor (with literature-reported values up to 1.04 mg/mL [71]). Given the excellent glucose-lowering effect of AVEO, it can serve as a natural alternative to hypoglycemic drugs.

Previous studies have examined the antioxidant capacity of water and extracts of *A. villosum*, particularly phenolic acids and flavonoids [17,18,72]. While plant phenolics are classical antioxidants [35], our in vitro experiments confirmed that AVEO also has good antioxidant capacity, with a maximum broad-spectrum scavenging rate of 84.59% against DPPH, ·OH, and ·O_2_^−^. Among them, the hydroxyl radical scavenging activity of Amomum4 (IC_50_ = 0.18 mg/mL) showed no statistical difference from reference standard vitamin C (IC_50_ = 0.14 mg/mL; *p* > 0.05), suggesting that AVEO could serve as a natural antioxidant to replace synthetic antioxidants.

Notably, correlation analysis showed a significant positive correlation between α-glucosidase inhibitory activity and DPPH scavenging capacity (r = 0.82), but a significant negative correlation with ·OH scavenging capacity (*r* = −0.65). This differential association may be related to the differences in the scavenging mechanisms of the two types of free radicals and their effects on enzyme active sites. DPPH clearance relies on electron transfer from hydroxyl or amino groups, potentially enhancing enzyme inhibition via active site interactions [73]. Conversely, ·OH scavenging depends on hydrogen atom donation [35], and strong ·OH scavengers may generate oxidative byproducts that impair enzyme activity [74]. These correlations highlight the functional synergy between hypoglycemic and antioxidant activities of AVEO; however, the mechanism underlying this functional synergistic effect requires further in-depth analysis.

### 4.3. Potential Hypoglycemic–Antioxidant Synergistic Mechanisms of AVEO

Network pharmacology and molecular docking identified AVEO components A4-A9 as key regulators of oxidative stress and glucose metabolism via multi-target synergy, with major targets and hub pathways mediating its hypoglycemic–antioxidant synergy through multi-dimensional cross-regulation (Figure 5, Figure 6 and Figure 7). Molecular docking further verified the high-affinity binding of (−)-spathulenol and (−)-pogostol to JAK2, NCOA2, and PTGS2, as well as the favorable affinity of bornyl acetate for PPARA (Figure 7). The PPAR, nuclear receptor-mediated steroid hormone, and JAK-STAT signaling pathways were identified as core regulators of AVEO antioxidant and hypoglycemic mechanisms in this study. For PPAR signaling pathway, it aligns with previously reported improvements in anti-diabetic efficacy linked to enhanced PPAR expression [75]. Type 2 diabetes mellitus (T2DM) rat models have demonstrated that hypoglycemic effects, insulin sensitization, antioxidant activity, and PPARA upregulation are associated [76,77,78]. In the present study, bornyl acetate, the most abundant component in AVEO (Figure 4), was also shown to have a favorable affinity for PPARA (−6.9 kcal/mol). While existing studies confirm its potent in vitro and in vivo antioxidant and anti-inflammatory activities [79,80], bornyl acetate’s direct role in hypoglycemia requires further validation. Given its dominance in composition and PPARA binding capability, bornyl acetate is hypothesized to be a primary contributor to AVEO’s observed antioxidant effects and potentially participates in hypoglycemic regulation through PPAR pathway modulation. Other components (e.g., (−)-spathulenol, (−)-pogostol, α-bisabolol) exhibited strong binding (binding energies < −7.2 kcal/mol with PPARA). These results suggest that AVEO may enhance insulin sensitivity or exert antioxidant effects through the activation of PPARα. Inhibition of the JAK-STAT pathway should be another core of the synergistic mechanism. As the inhibitory effect is recognized as one of the key mechanisms underlying metformin, a first-line anti-diabetic drug [81], previous studies have indicated that normalization or downregulation of the JAK-STAT pathway improves diabetic phenotypes and increases anti-inflammatory cytokine levels [82]. The significant enrichment of AVEO’s antioxidant–hypoglycemic intersection targets in the JAK-STAT pathway further supports the theory of multi-target synergistic regulation through this pathway. All components A1–A9 showed potential for stable binding to JAK2, with (−)-spathulenol (binding energy: −9.0 kcal/mol) and (−)-pogostol (binding energy: −8.2 kcal/mol) showing the most stable binding. For other pathways, the PI3K/AKT pathway is considered a critical therapeutic target for obesity and T2DM [76,77,78]. However, neither GO nor KEGG analyses revealed significant enrichment of this pathway, offering no direct support for Hypothesis 3. Nevertheless, several identified targets may still modulate PI3K/AKT activity. Nonetheless, other target proteins could directly affect the PI3K/AKT pathway. For instance, JAK2 phosphorylation [83,84], improvement in PTPN1 (PTP1B) [85,86], or through nuclear receptor-mediated steroid hormone signaling [87] could influence the pathway. In this study, most active components (bornyl acetate, (−)-spathulenol, (−)-pogostol, α-bisabolol) were found to have the potential for stable binding to targets JAK2 and PTPN1 (Figure 7), with the nuclear receptor-mediated steroid hormone signaling pathway also identified among the major enriched pathways. These findings collectively indicate that AVEO modulates the PI3K/AKT pathway through parallel mechanisms. Thus, the results substantiate the mechanism whereby these core components (e.g., bornyl acetate, (−)-spathulenol, and (−)-pogostol) mitigate hyperglycemia and oxidative stress by regulating pivotal targets (e.g., CYP3A4, PPARA, JAK2) to activate a multidimensional network involving (e.g., the PPAR signaling pathway, nuclear receptor-mediated steroid hormone pathway, and JAK-STAT signaling pathway).

Elucidation of binding sites provides a structural basis for the aforementioned mechanisms. Research on the stable binding bonds between AVEO and core targets related to hypoglycemic and oxidative stress effects is limited. A previous study reported that a compound from the stems and leaves of *A. villosum* [(7S-9′R)-(4,5-dihydroxy-3-methoxyphenyl)-7-methoxyacetoxy)-7′-(1′-hydroxyphenyl)butanoic acid] formed hydrogen bond interactions with Lys93 and Agr214 to maintain binding with the antioxidant target protein PGAM5, thereby inhibiting the upregulation of intracellular reactive oxygen species levels under oxidative stress stimulation [26]. Conversely, using the fruit extract, we identified distinct targets and binding modes. The most stably bound compounds, (−)-spathulenol and (−)-pogostol targeted NCOA2, JAK2, CYP3A4, and CYP2C19 through differential hydrogen bonds (e.g., Ser936) and hydrophobic interactions (e.g., Tyr930, LEU855, LEU982, LEU983, ALA880, VAL863, LEU346, LEU349, LEU384, LEU387, LEU525, ALA350, TRP383, PHE404). Components with relatively high content targeted PPARA through hydrogen bonds (e.g., Asn219, Met220) and hydrophobic contacts (e.g., Leu321, Val324). To some extent, this provides new binding site insights for the regulation of hypoglycemic and antioxidant effects by AVEO.

### 4.4. Limitations and Future Perspectives

Our study elucidates the material basis and molecular network underlying the synergistic antioxidant and hypoglycemic effects of AVEO through an “extraction optimization–functional validation–mechanistic elucidation” framework. Predictive conclusions from network pharmacology and molecular docking require experimental validation, representing an essential progression for in-depth mechanistic exploration. Future work will proceed from computational prediction to experimental verification to functional food development continuum, comprehensively evaluating AVEO’s hypoglycemic and antioxidant effects through integrated cellular and animal studies. This will provide more robust experimental evidence for applying *A. villosum* in metabolic disorder management and functional foods.

## 5. Conclusions

An optimized ultrasound-assisted extraction protocol for AVEO was established in this study. The actual extraction conditions (15 min ultrasonication, 1:26.13 g/mL solvent-to-material ratio, and 200 W power) were determined using response surface methodology, yielding a high extraction rate of 3.84%. AVEO exhibited concentration-dependent α-glucosidase inhibitory activity, with efficacy comparable to the reference substance, acarbose. AVEO also demonstrated potent scavenging activity against DPPH, ·OH, and ·O_2_^−^ radicals. Mechanistic studies indicated that the dual bioactivities involve shared bioactive constituents. Network pharmacology analysis identified terpinen-4-ol, α-terpineol, bornyl acetate, (‒)-spathulenol, (‒)-pogostol, and α-bisabolol as core components that regulate a multi-target network encompassing CYP3A4, CYP2C19, PTGS2, NR3C1, PPARA, NCOA2, JAK2, and ESR1. PPI analysis revealed that these targets are significantly enriched in the PPAR signaling pathway, steroid hormone pathway, and JAK-STAT cascade. Molecular docking validated the strong binding abilities of (‒)-spathulenol and (‒)-pogostol to JAK2 and NCOA2 (binding energy < −8.2 kcal/mol), with stable binding involving hydrogen bonds and hydrophobic interactions. Additionally, the strong binding potential of the main component, bornyl acetate, to PPARA via hydrogen bonds and hydrophobic interactions was confirmed. Collectively, these results elucidate the synergistic multi-target mechanisms underlying the antioxidant and hypoglycemic efficacy of AVEO, laying a foundation for the development of nutraceuticals and therapeutic agents derived from *Amomum villosum*. However, further cellular and in vivo studies are needed to assess its therapeutic efficacy and safety profile fully.

## Figures and Tables

**Table 1:** *Amomum villosum* sample information.

Sample	Collection Site	Planting Year	Collection Year	Planting Density (Plants/m^2^)	Annual Output (kg/ha)
Amomum1	Leyi Village, Wangdian Yao Ethnic Township, Youjiang District, Baise City, Guangxi, China	2019	2023	8.60 ± 0.55 a	1233.30 ± 60.00 a
Amomum2		2019	2022	8.20 ± 0.45 a	1224.60 ± 149.90 a
Amomum3	Shangmeng Village, Pingshan Township, Long’an County, Nanning City, Guangxi, China	2019	2023	8.80 ± 0.84 a	1248.00 ± 106.00 a
Amomum4		2019	2022	8.60 ± 1.14 a	1319.40 ± 143.20 a

The letter “a” indicates no significant differences (*p* > 0.05) among Amomum1, Amomum2, Amomum3, and Amomum4 groups based on the LSD test.

**Table 2:** Analysis of variance results from the response surface test. Statistical significance of linear, quadratic, and interaction terms for extraction variables affecting AVEO (*A. villosum* essential oil) yield.

Source of Variation	Sum of Squares	Degrees of Freedom (df)	Mean Square	*F*	*p*
Model	2.02	9	0.2249	13.89	0.0011 **
A—Ultrasound Time	0.0005	1	0.0005	0.0278	0.8723
B—Solvent-to-Material Ratio	0.0925	1	0.0925	5.71	0.0482 *
C—Ultrasound Power	0.0085	1	0.0085	0.522	0.4934
AB	0.0552	1	0.0552	3.41	0.1072
AC	0.042	1	0.042	2.6	0.1512
BC	0.0182	1	0.0182	1.13	0.3239
A^2^	0.6104	1	0.6104	37.71	0.0005 ***
B^2^	0.2543	1	0.2543	15.71	0.0054 **
C^2^	0.7632	1	0.7632	47.15	0.0002 ***
Residual	0.1133	7	0.0162		
Lack of Fit	0.01	3	0.0033	0.129	0.9379
Pure Error	0.1033	4	0.0258		
Total	2.14	16			
R^2^ = 0.9470	R^2^_adj_ = 0.8788				

* *p* < 0.05, ** *p* < 0.01, *** *p* < 0.001.

**Table 3:** Gas chromatography–mass spectrometry (GC-MS) profile of AVEO. Identified chemical constituents are listed with their retention times and relative contents of key active ingredients based on GC–MS analysis.

No.	Retention Time (min)	Component	Molecular Formula	CAS	Match Factor (NIST20.L,%)	Relative Peak Area (%)	GI Absorption	No. of Active Ingredients
1	8.457	p-Cymene	C10H14	99-87-6	87.06	0.35	Low	/
2	8.74	Cyclohexane-1-butenylidene-	C10H16	36144-40-8	86.49	5.21	Low	/
3	10.42	Linalool	C10H18O	78-70-6	87.37	0.59	High	A1
4	11.463	(+)-2-Bornanone	C10H16O	464-49-3	88.93	15.91	High	A2
5	11.907	Isoborneol	C10H18O	124-76-5	86.13	0.16	High	A3
6	12.169	5,5-Dimethyl-1,3-hexadiene	C8H14	1515-79-3	86.78	3.52	Low	/
7	12.495	Terpinen-4-ol	C10H18O	562-74-3	85.85	0.43	High	A4
8	12.811	α-Terpineol	C10H18O	98-55-5	86.15	0.19	High	A5
9	15.71	Bornyl acetate	C12H20O2	5655-61-8	87.68	52.1	High	A6
10	18.785	(+)-Cyclosativene	C15H24	22469-52-9	88.06	1.43	Low	/
11	22.422	α-Cubebene	C15 H24	17699-14-8	91.85	0.25	Low	/
12	24.075	β-Sesquiphellandrene	C15H24	20307-83-9	86.66	0.66	Low	/
13	25.974	(−)-Spathulenol	C15H24O	77171-55-2	86.82	0.8	High	A7
14	26.691	(−)-Pogostol	C15H26 O	21698-41-9	93.13	0.18	High	A8
15	30.681	α-Bisabolol	C15H26 O	515-69-5	85.83	0.16	High	A9

The query results for gastrointestinal (GI) absorption were sourced from the SwissADME database (http://www.swissadme.ch/index.php, accessed on 30 May 2025). Components marked as “high” for GI absorption were selected as active ingredients.

## Data Availability

The original contributions presented in this study are included in the article. Further inquiries can be directed to the corresponding author.

## Supplementary Materials

The following supporting information can be downloaded at https://www.mdpi.com/article/10.3390/foods14162772/s1: Table S1: Box–Behnken design and results for response surface optimization. Design matrix and corresponding outcomes for ultrasonic extraction parameters affecting essential oil yield from AVEO.

## Abbreviations

The following abbreviations are used in this manuscript:

AVEO	*Amomum villosum* essential oil
α-GI	α-Glucosidase inhibition
DPPH	2,2-Diphenyl-1-picrylhydrazyl
GC-MS	Gas chromatography–mass spectrometry
GO	Gene ontology
KEGG	Kyoto Encyclopedia of Genes and Genomes
PPI	Protein-protein interaction
RNA	Ribonucleic acid
DNA	Deoxyribonucleic acid
PPAR	Peroxisome proliferator-activated receptor
JAK-STAT	Janus kinase–signal transducer and activator of transcription
TIC	Total ion chromatogram
