# Supplementary material for: Ultrasound-Optimized Extraction and Multi-Target Mechanistic Analysis of Antioxidant and Hypoglycemic Effects of Amomum villosum Essential Oil
Source: Foods. 2025 Aug 9;14(16):2772. doi: 10.3390/foods14162772 (PMC12385424; doi:10.3390/foods14162772)
Supplement: Supplementary file 1 [file foods-14-02772-s001.zip › foods-3747331-supplementary.pdf]

Table S1. Box-Benhnken design and results for response surface optimization. Design matrix and corresponding outcomes for ultrasonic extraction parameters affecting essential oil yield from *Amomum villosum* essential oil (AVEO).

No.	Ultrasound Time (min)	Solvent-to-Material Ratio (g/mL)	Ultrasound Power (W)	essential Oil (mL)	Extraction Rate (%)
1	10	1:20	200	0.44	2.93
2	20	1:20	200	0.48	3.20
3	10	1:30	200	0.5	3.33
4	20	1:30	200	0.47	3.13
5	10	1:25	160	0.46	3.07
6	20	1:25	160	0.42	2.80
7	10	1:25	240	0.44	2.93
8	20	1:25	240	0.46	3.07
9	15	1:20	160	0.43	2.87
10	15	1:30	160	0.49	3.27
11	15	1:20	240	0.46	3.07
12	15	1:30	240	0.48	3.20
13	15	1:25	200	0.54	3.60
14	15	1:25	200	0.56	3.73
15	15	1:25	200	0.58	3.87
16	15	1:25	200	0.60	4.00
17	15	1:25	200	0.55	3.67
